# Distinguishing three Dragon fruit (*Hylocereus* spp.) species grown in Andaman and Nicobar Islands of India using morphological, biochemical and molecular traits

**DOI:** 10.1038/s41598-021-81682-x

**Published:** 2021-02-03

**Authors:** K. Abirami, S. Swain, V. Baskaran, K. Venkatesan, K. Sakthivel, N. Bommayasamy

**Affiliations:** ICAR- Central Island Agricultural Research Institute, Port Blair, Andaman and Nicobar Islands 744 101 India

**Keywords:** Biochemistry, Biotechnology, Molecular biology, Physiology, Plant sciences

## Abstract

Dragon fruit (*Hylocereus* spp.), an important tropical fruit belonging to the family Cactaceae, is rich in essential nutrients such as vitamins, minerals, complex carbohydrates, dietary fibres and antioxidants. This study aims to distinguish three dragon fruit species well adapted to Andaman and Nicobar Island through morphological (34 quantitative and 26 qualitative traits), biochemical (5 traits) and molecular (14 ISSR primers) characterization. Morphological characterization revealed that presence of considerable amount of genetic variations among them especially for fruit characters viz., colour of peel and pulp. Cladode characters such as number of spines (3–5), length of areoles (mm) as 1–4, margin ribs of cladode (convex or concave) and its waxiness (weak or strong white waxy or light waxy) could be used for identification of three *Hylocereus* spp. under present study. Highest co-efficient of variation (%) obtained for pulp weight (88.7), whereas, lowest in distance of anthers belowstigma (3.3). Fruit and pulp weight (g) ranged from 26.5–419.3 and 10.3–258.8 with mean value of 204.8 and 125.3, respectively. Comparatively, high phenol (71.3–161.3) and flavonoid (26.6–508.2) content observed in peels than pulp (32.5–130.0 and 45.0–258.2) of fruit indicating higher antioxidant potential. Highest total carotenoids (µg 100 g^−1^), β-carotene (µg 100 g^−1^) and xanthophyll (µg g^−1^) content obtained in pulp on DGF3 (33.8), DGF4 (55.9) and DGF3 (32.7), whereas, in peel on DGF2 (24.3), DGF4 (18.5) and DGF2 (24.1), respectively. DPPH-based scavenging activity (%) revealed higher scavenging activity of peels (55.6–81.2) than pulp (36.0–75.3) extracts. Comparatively, ABTS-based scavenging activity (%) was found more than DPPH-based one. Sixteen ISSR primers screened, 14 were produced 178 reproducible amplified bands. Number of amplified bands varied from 5 in UBC887 to 19 in UBC811 with an average of 12.71 bands per primer. Range of polymorphic bands and % polymorphism observed were 1–13 and 20.0–92.8, respectively. The polymorphic information content value of ISSR marker ranged from 0.42 (UBC895) to 0.91 (UBC 856). Cluster analysis distinguished three different *Hylocereus* species on the basis of geographic origin and pulp colour by forming separate groups and two genotypes each showed 52% (DGF1 and DGF3) and 76% (DGF2 and DGF4) genetic similarity. Key traits identified for distinguishing three different *Hylocereus* species were: Pulp/ peel colour of fruits, number of spines and length of areoles in cladode. Genotypes with high carotenoid and xanthophylls content (DGF4 and DGF2) identified under present study may be of industrial importance for development of nutraceutical products to meet out the vitamin-A deficiency among humans in tropical regions needed future focus.

## Introduction

Dragon fruit (*Hylocereus* spp.), also known as Pitaya or Pitahaya belonging to the family Cactaceae, is an most important tropical fruit crop as it is rich in antioxidants (a source of vitamins and minerals, prevent cancer, diabetes, cardiovascular, respiratory, gastrointestinal and urinary diseases), as well as dietry fiber and low in calories^1,2^. It has drawn worldwide attention owing to its new flavour, colour and attractive appearance along with their enormous health benefits. It gained great commercial potential in India by consumer preference for new and exotic phyto-chemically rich nutrient fruits and its adaptability to new environment with abiotic stress tolerance like drought and temperature extremes^3^. Although 14 *Hylocereus* spp. reported in worldwide^4^, only four species such as *H. undatus*, *H. monocanthus* (Syn. *H. polyrhizus*), *H. costariscensis* and *H. megalanthus* (Syn. *Selenicereus megalanthus*) are mostly cultivated in different parts of the world^5^. Among them, the *H. undatus* is widely cultivated in Indian States like Tamil Nadu, Andhra Pradesh, Karnataka, Maharashtra, Punjab and West Bengal.

In general, fresh fruits and vegetables are excellent source of antioxidant constituents (phenols and flavanoids) which confer protection against chronic diseases caused by oxidative stress, namely cardio-vascular disorders and some kinds of cancer. Recently, dragon fruit cultivation was introduced in Andaman and Nicobar Islands which is highly dependent on import of common fruits and vegetables from mainland India to meet out nutrient requirement of Islands. This fruits are also known for its richness in antioxidant and antiproliferation properties^6–8^ with phenolic and polyphenolic compounds^2,6,7^.

Nowadays, it is very difficult to separate species and varieties of the dragon fruit owing to high intra and inter-specific hybridization which created some taxonomical confusion in worldwide^9^. Morphological and genetic heterogeneity in many fruits characteristics such as sweetness, size, shape, color, and bracts number by this intra and inter-specific hybridization^10,11^ makes it difficult to increase the quality standards for the exportation market^10^ because it posed serious problems in determining their performance in handling and shelf life.

Conventionally, morphological traits had been used to differentiate plant germplasm/ species and to elucidate their genetic relationship^12–15^. Though there were distinct morphological differences exists in stem, flower and fruit characters in this genus *Hylocereus*^7^, molecular characterization of them will help in elucidating the genetic relationship and diversity among the genotypes^16,17^. Keeping this in view, the present study was undertaken with the following objectives: (1) Morphological characterization of three introduced dragon fruit (*Hylocereus* spp.) species using quantitative and qualitative traits, (2) Molecular characterization of three dragon fruit (*Hylocereus* spp.) species using five biochemical traits including fruit colour values and ISSR marker, (3) Distinguishing three dragon fruit (*Hylocereus* spp.) species using morphological and molecular traits and (4) Identification of suitable species with rich antioxidants for development of nutraceutical products.

## Results

### Morphological characterization

**Figure 1:**
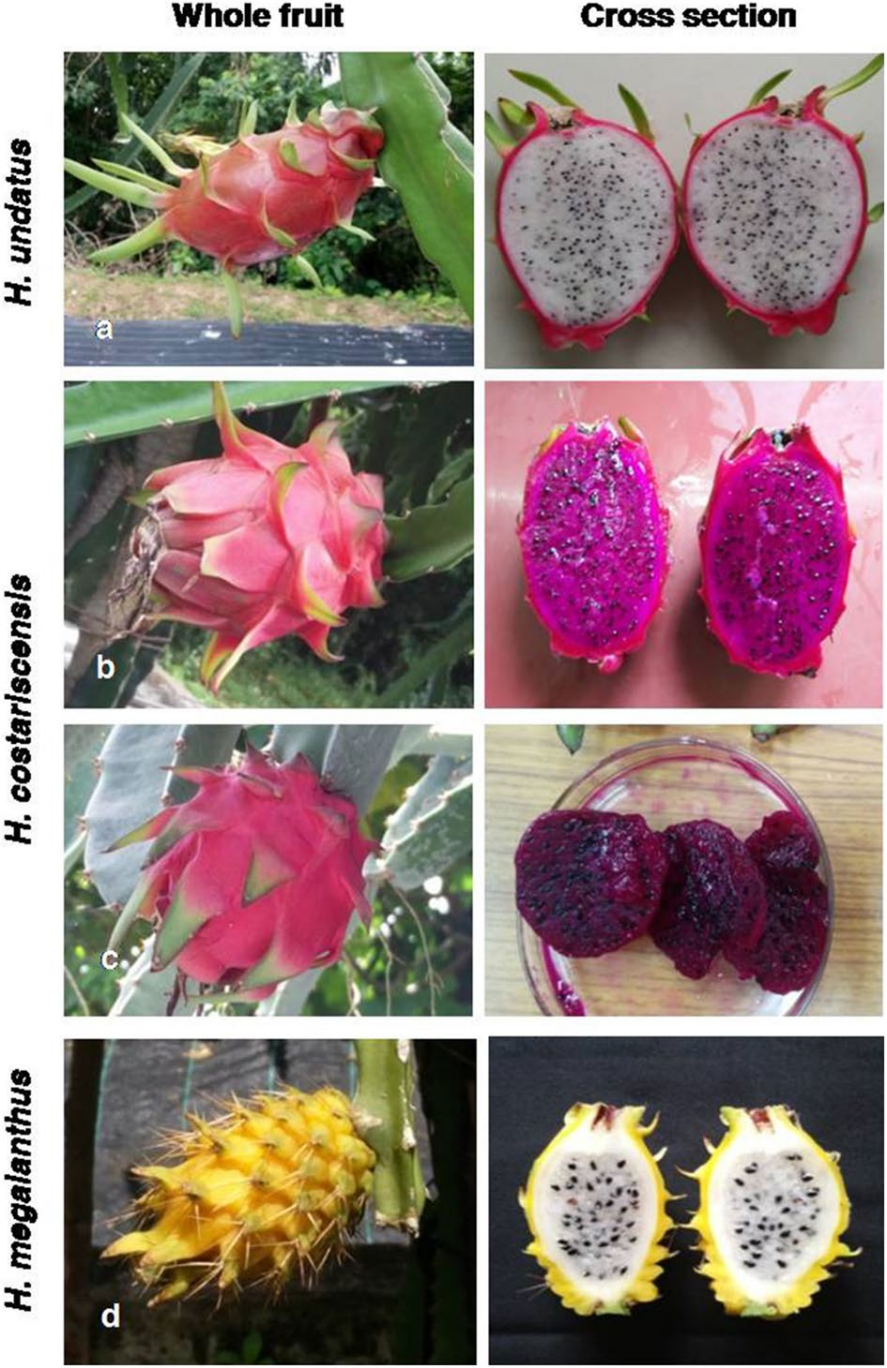
A characteristic fruit view of three different *Hylocereus* species of dragon fruit (1) *H. undatus*—DGF1 (**a**); (2) *H. costariscensis*—DGF2 (**b**), DGF4 (**c**) and (3) *H. megalanthus*—DGF3 (**d**).

**Figure 2:**
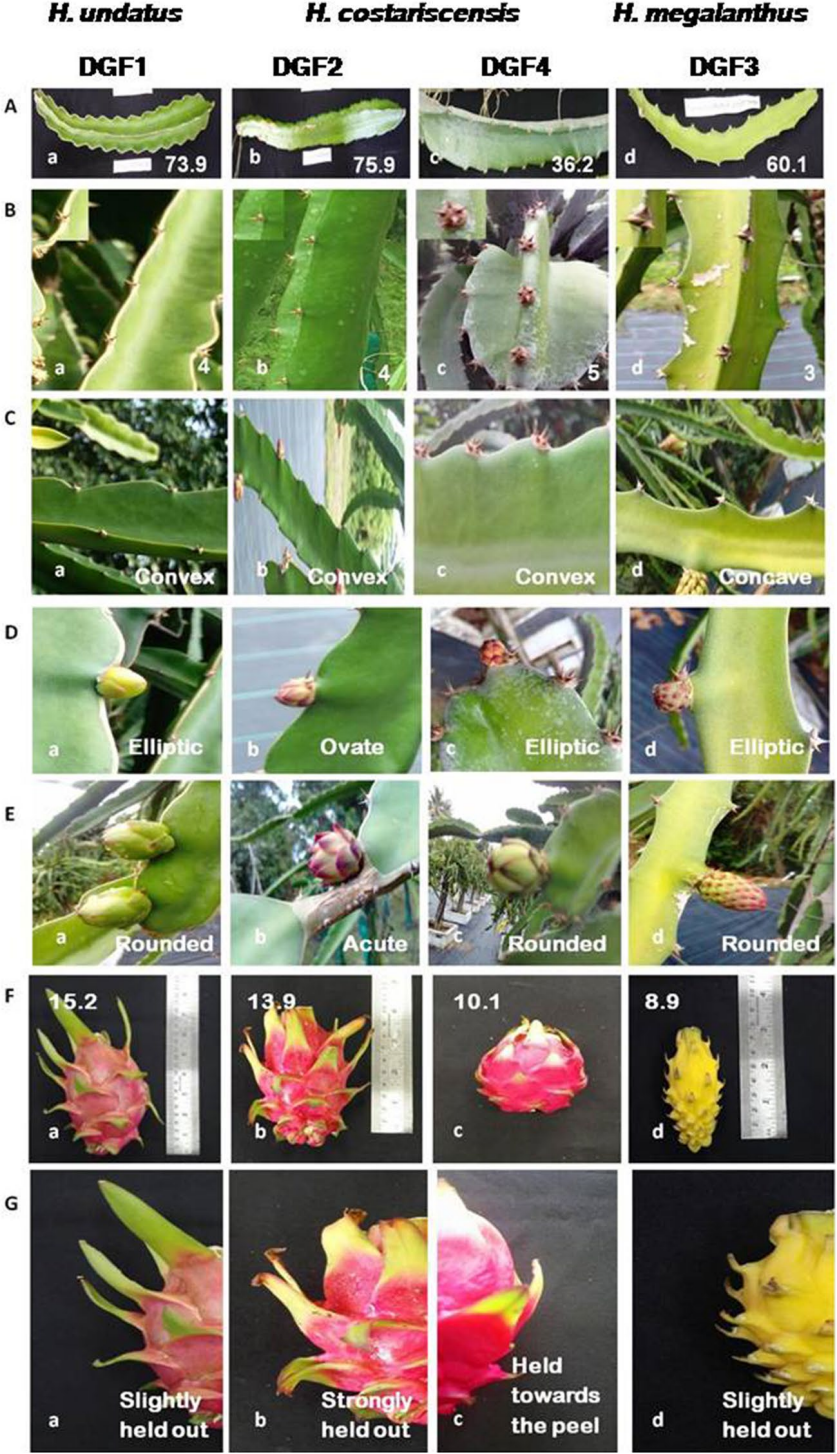
An illustration of important traits of three different *Hylocereus* species of dragon fruit. Cladode characters: (**A**) length of segments (cm), (**B**) number of spines and (**C**) margin ribs of cladode; Flower characters: (**D**) flower bud shape and (**E**) shape of apex; fruit characters: (**F**) fruit length (cm) and (**G**) position towards the peel.

Data recorded on 34 quantitative and 26 qualitative traits in four genotypes of three dragon fruit (*Hylocereus* spp.) species were presented in Tables 1 and 2. A characteristic view of three different *Hylocereus* species of dragon fruit on key traits of cladode, floral and fruit is presented in Figs. 1 and 2. Range of variation for dragon fruit (*Hylocereus* spp.) species using 34 quantitative traits under study is presented in Table 3. The highest co-efficient of variation (CV in %) was obtained for pulp weight (88.7) followed by fruit weight (85.3), number of fruiting cycles (78.0), average fruit yield plant^−1^ season^−1^ (70.5) and length of areoles (mm) (66.7), whereas, lowest observed in distance of anthers below stigma (mm) (3.3). Range of important cladode characters such as length of areoles (mm), arch height of cladode (mm), cladode width (mm), distance between areoles (mm) and number of spines varied from 1–4, 2–6, 35–53, 34–40 and 3–5 with mean value of 2.3, 3.5, 41.3, 36.8 and 4.0, respectively. Flower and its style length ranged from 18.4–23.2 and 7.0–13.4 with mean value of 20.7 and 10.2, respectively, whereas, average number of flowers plant^−1^ fruiting cycle^−1^ ranged from 10.3 to 15.8 with mean value of 12.5. Among fruit characters, fruit and pulp weight ranged from 26.5–419.3 and 10.3–258.8 with mean value of 204.8 and 125.3, respectively. In case of number of bracts and length of apex bract in fruit varied from 27–59 and 2.1–6.2 with mean value of 39.0 and 4.0, respectively. Range and mean value of fruit yield (kg plant^−1^ season^−1^) obtained as 1.1–6.3 and 3.6, respectively.

**Table 1:** Characterization of three dragon fruit (*Hylocereus*) species with 34 quantitative traits of cladode, flower and fruit in Andaman and Nicobar Islands.

Accession/descriptors	*H. undatus*	*H. costariscensis*		*H. megalanthus*
	DGF 1	DGF 2	DGF 4	DGF 3
**Cladode**				
Length of segments (cm)	73.9	75.9	36.2	60.1
Cladode width (mm)	39	38	53	35
Arch height of cladode (mm)	6	4	2	2
Distance between areoles (mm)	38	35	34	40
Number of spines	4	4	5	3
Length of areoles (mm)	3	4	1	1
**Flower**				
Flower bud length (mm)	25	26	25	21
Flower bud width (mm)	12	14	16	9
Pericarpel length (mm)	205	243	246	246
Pericarpel width (mm)	37	36	37	32
Flower pericarpel: separation of bracts	7.58	8.46	7.16	8.12
Flower length (cm)	23.22	19.26	18.36	22.15
Flower: length of style (cm)	13.42	7.34	7.02	12.89
Flower: number of stigma lobes	28.2	23.4	20.3	28.1
Flower bifurcation of stigma lobes (cm)	2.18	1.76	1.67	2.13
Distance of anthers below stigma (mm)	15	15	15	16
Days to bud initiation (DFT)	457.8	422.5	863.8	845.6
Days to anthesis	30.6	29.8	33.5	37.3
Average no. of flowers plant^−1^ fruiting cycle^−1^	11.3	12.5	10.3	15.8
**Fruit**				
Fruit length (cm)	15.2	13.9	10.1	8.9
Fruit diameter (cm)	10.9	9.6	9.3	4.2
Total fruit weight (g)	419.3	267.4	106	26.5
Peel weight (g)	160.5	97.3	45.6	16.3
Pulp weight (g)	258.8	170.1	61.9	10.3
No. of bracts	37	33	27	59
Length of the apex bracts (mm)	62	29	47	21
Width of the base of the bract (mm)	47	32	23	8
Distance between bract to bract (mm)	51	37	41	10
TSS ( ^o^B)	11.2	15.9	9.1	18.3
Titrable acidity (%)	0.22	0.19	0.28	0.16
Average fruit set fruiting cycle^−1^ plant^−1^ (%)	83.8	63.9	44.9	41.3
Days from anthesis to fruit maturity	19.3	22.5	18.7	25.6
Number of fruiting cycles	07	08	02	01
Average fruit yield season^−1^ plant^−1^ (Kg)	6.3	5.2	1.8	1.1

**Table 2:** Characterization of three dragon fruit (*Hylocereus*) species with 26 qualitative traits of cladode, flower and fruit in Andaman and Nicobar Islands.

Accession/descriptors	*H. undatus*	*H. costariscensis*		*H. megalanthus*
	DGF 1	DGF 2	DGF 4	DGF 3
**Cladode**				
Margin ribs of cladode	Convex	Convex	Convex	Concave
Cladode waxiness	Weak	Weak	Strong white wax	Light waxy
Young cladode colour	Light reddish	Light reddish	Light reddish	Light reddish
**Flower**				
Flower bud shape	Elliptic	Ovate	Elliptic	Elliptic
Shape of apex	Rounded	Acute	Rounded	Rounded
Flower bud main colour	Yellowish green	Yellowish red	Greenish purple	Yellow with pink tinge
Flower pericarpel: reddish colour intensity of bracts	Green with purple edge	Green with dark red edge	Green with dark pink edge	Dark Green with light pink edge
Sepal pattern	Edged	Edged	Edged	Edged
Shape of bracts	Ovate	Ovate	Ovate	Ovate
Flower colour of petals	Milky white	Milky white	Milky white	Milky white
Flower colour of sepals	Light green	Light green	Light green	Green
Flower colour pattern of sepals	Greenish with purple edge	Lemon yellow	Greenish with purple edge	Greenish yellow
Flower colour of stigma lobe	Creamish green	Cream	Cream	Creamish green
Flower colour of ring at base of reproductive organs	Lemon yellow with purple edge	Lemon yellow with dark red edge	Lemon yellow with red edge	Greenish yellow with pink edge
Month of bud initiation	April	March	May	May
Time of anthesis	11.00 PM	11.00 PM	11.30 PM	10.30 PM
Duration of anthesis	4–6 h	4–6 h	4–6 h	4–6 h
**Fruit**				
Fruit shape	Moderately elongated	Medium elongated	Moderately rounded	Elongated
Fruit width	Narrow	Broad	Medium	Narrow
Position towards the peel	Slightly held out	Strongly held out	Held towards the peel	Slightly held out
Colour of pulp	White	Pink	Dark Purple	White
Colour of peel	Pinkish green	Pinkish Red	Pink	Yellow
Juiciness of flesh	Medium	High	Medium	High
Seed size	Small	Medium	Medium	Large
No. of seeds fruit^−1^	Many	Many	Many	Medium
Fruiting season	May to Oct	Apr to Nov	Jun to Sept	Jul to Sept

**Table 3:** Range of variation for 34 quantitative traits of three dragon fruit (*Hylocereus*) species of cladode, flower and fruit in Andaman and Nicobar Islands.

Descriptors	Min	Max	Mean	SE	SD	Skewness	Kurtosis	CV
**Cladode**								
Length of segments (cm)	36.2	75.9	61.5	9.1	18.3	− 1.2	0.7	29.7
Cladode width (mm)	35.0	53.0	41.3	4.0	8.0	1.7	3.2	19.4
Arch height of stem (mm)	2.0	6.0	3.5	1.0	1.9	0.9	− 1.3	54.7
Distance between Areoles (mm)	34.0	40.0	36.8	1.4	2.8	0.3	− 3.0	7.5
Number of spines	3.0	5.0	4.0	0.4	0.8	− 6.9	1.5	20.5
Length of Areoles (mm)	1.0	4.0	2.3	0.8	1.5	0.4	− 3.9	66.7
**Flower**								
Flower bud length (mm)	21.0	26.0	24.3	1.1	2.2	− 1.7	3.3	9.1
Flower bud width (mm)	9.0	16.0	12.8	1.5	3.0	− 0.4	− 0.4	23.4
Pericarpel length (mm)	205.0	246.0	235.0	10.0	20.0	− 2.0	3.9	8.5
Pericarpel width (mm)	32.0	37.0	35.5	1.2	2.4	− 1.8	3.1	6.7
Flower pericarpel: separation of bracts	7.2	8.5	7.8	0.3	0.6	− 0.1	− 2.2	7.3
Flower length (cm)	18.4	23.2	20.7	1.2	2.3	0.1	− 4.3	11.1
Flower: length of style (cm)	7.0	13.4	10.2	1.7	3.5	0.0	− 5.8	34.0
Flower: number of stigma lobes	20.3	28.2	25.0	1.9	3.9	− 0.5	− 3.0	15.4
Flower bifurcation of stigma lobes (cm)	1.7	2.2	1.9	0.1	0.3	− 0.1	− 5.2	13.3
Distance of anthers below stigma (mm)	15.0	16.0	15.3	0.3	0.5	2.0	4.0	3.3
Days to bud initiation (DFT)	422.5	863.8	647.4	119.9	239.9	0.0	− 5.9	37.1
Days to anthesis	29.8	37.3	32.8	1.7	3.4	0.9	− 0.6	10.4
Average no. of flowers plant^−1^ fruiting cycle^−1^	10.3	15.8	12.5	1.2	2.4	1.2	1.4	19.2
**Fruit**								
Fruit length (cm)	8.9	15.2	12.03	15.0	30.0	0.0	− 4.4	25.0
Fruit diameter (cm)	4.2	10.9	8.5	14.7	29.5	− 1.7	3.1	34.7
Total fruit weight (g)	26.5	419.3	204.8	87.3	174.6	0.4	− 1.9	85.3
Peel weight (g)	16.3	160.5	79.9	31.6	63.3	0.6	− 1.0	79.2
Pulp weight (g)	10.3	258.8	125.3	55.6	111.2	0.3	− 2.3	88.7
No. of bracts	27.0	59.0	39.0	7.0	14.0	1.5	2.5	35.8
Length of the apex bracts (mm)	21.0	62.0	39.8	9.2	18.4	0.4	− 2.3	46.3
Width of the base of the bract (mm)	8.0	47.0	27.5	8.2	16.3	0.0	0.1	59.4
Distance between bract to bract (mm)	10.0	51.0	34.8	8.8	17.5	− 1.3	2.3	50.4
TSS ( ^o^B)	9.1	18.3	13.6	2.1	4.2	0.1	− 3.4	31.0
Titrable acidity (%)	0.2	0.3	0.2	0.0	0.1	0.8	0.3	24.1
Average fruit set fruiting cycle^−1^ plant^−1^ (%)	41.3	83.8	58.5	9.8	19.6	0.8	− 1.4	33.5
Days from anthesis to fruit maturity	18.7	25.6	21.5	1.6	3.2	0.7	− 1.7	14.8
Number of fruiting cycles	1.0	8.0	4.5	1.8	3.5	0.0	− 5.2	78.0
Average fruit yield season^−1^ plant^−1^ (Kg)	1.1	6.3	3.6	1.3	2.5	0.1	− 4.7	70.5

### Biochemical characterization

**Figure 3:**
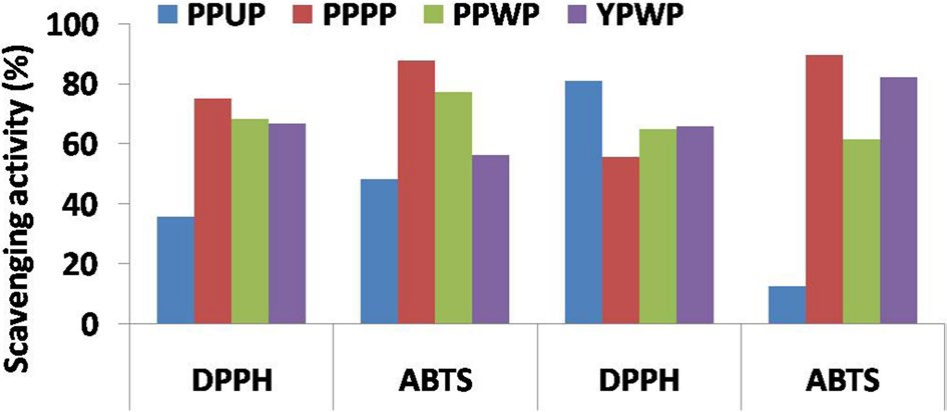
Antioxidant activity (DPPH and ABTS method) in pulp and peel of four different dragon fruit accessions [PPUP (pink peel with dark purple pulp) = DGF4; PPPP (pinkish red peel with pink pulp) = DGF2; PPWP (pinkish green peel with white pulp) = DGF1; YPWP (yellow peel with white pulp) = DGF3]; ^*^DGF1 = *Hylocereus undatus*; DGF2 & DGF4 = *Hylocereus costariscensis*; DGF3 = *Hylocereus megalanthus*.

Biochemical characterization of four genotypes of three dragon fruit (*Hylocereus* spp.) species with total phenol content (TPC), total flavonoid content (TFC), total carotenoid content (TCC), β-carotene, xanthophyll and colour values such as L, a, b, hue and chroma were presented in Table 4. Among four genotypes, DGF4 had highest phenolic content (mg GAE 100 g^−1^) in both peel (161.3) and pulp (130.0) extracts followed by DGF3 (118.8 and 103.8), DGF1 (42.5 and 71.3) and DGF2 (32.5 and 91.3) in pulp and peel extracts, respectively, with the coefficient of variation (CV) of 0.62. Highest flavonoid content (mg RE 100 g^−1^) was found in peel of DGF4 (508.2) followed by DGF3 (123.9), DGF1 (55.5), whereas lowest in DGF2 (26.6). In case of pulp, it was varied from 45.0 (DGF1) to 258.2 (DGF4). DGF3 pulp showed highest (33.8) carotenoids content (µg 100 g^−1^) followed by DGF2 (30.4) and DGF4 (30.0) with the CV of 0.24, whereas, in peel highest and lowest obtained on DGF2 (24.3) and DGF1 (4.82), respectively. The highest content of β-carotene (µg 100 g^−1^) was found in DGF4 (55.9 and 18.5) and DGF2 (53.2 and 16.4) than DGF3 (1.3 and 0.9) and DGF1 (1.2 and 0.2) in pulp and peel, respectively. Xanthophyll content (µg g^−1^) of DGF3, DGF2 and DGF4 pulp was found as 32.7, 29.8 and 29.5, respectively, whereas, in peel it was varied from 4.8 (DGF1) to 24.1 (DGF2). The scavenging activity (%) by DPPH and ABTS method varied between 36.0 (DGF4) to 75.3 (DGF2) and 48.3 (DGF4) to 87.9 (DGF2), respectively, in pulps and, 55.6 (DGF2) to 81.2 (DGF4) and 61.8 (DGF1) to 89.8 (DGF2), respectively, in peels of four genotypes with CV of 0.29 and 0.26, respectively (Fig. 3). The observed colour values of four dragon fruit genotypes were as: L (11.7–51.0 and 13.6–40.8), a (− 0.6 to 21.4 and 3.8 to 24.0) and b (− 2.5 to 2.5 and 4.4 to 19.0) in pulp and peel, respectively. In pulp, hue and chroma values varied from − 76.0 to 77.5 and 2.1 to 21.5 respectively, whereas, it varied from 16.3 to 78.5 and 12.0 to 26.0 respectively, in peel of dragon fruits.

**Table 4:** Biochemical parameters including colour values of three dragon fruit (*Hylocereus*) species under Andaman and Nicobar Island conditions of India.

Parameters/genotype	Pulp				Peel				Range		Mean		C.V
	DGF1*	DGF2	DGF3	DGF4	DGF1	DGF2	DGF3	DGF4	Pulp	Peel	Pulp	Peel	
**Phenol**													
TPC**	42.5	32.5	118.8	130.0	71.3	91.3	103.8	161.3	32.5–130.0	71.3–161.3	80.9	106.9	0.62
**Flavonoid**													
TFC	45.0	147.6	102.9	258.2	55.5	26.6	123.9	508.2	45.0–258.2	26.6–508.2	138.4	178.5	0.65
**Carotenoid**													
TCC	18.5	30.4	33.8	30.0	4.8	24.3	9.9	23.7	18.5–33.8	4.8–24.3	28.2	15.7	0.24
β-Carotene	1.2	53.2	1.3	55.9	0.2	16.4	0.9	18.5	1.2–55.9	0.2–18.5	27.9	9.0	1.10
Xanthophyll	18.5	29.8	32.7	29.5	4.8	24.1	9.9	23.5	18.5–32.7	4.8–24.1	27.7	15.6	0.23
**Antioxidant**													
By DPPH (%)	70.1	75.3	70.0	36.0	65.0	55.6	65.9	81.2	36.0–75.3	55.6–81.2	62.8	66.9	0.29
By ABTS (%)	68.1	87.9	56.4	48.3	61.8	89.8	82.4	86.4	48.3–87.9	61.8–89.8	65.2	80.1	0.26
**Colour**													
L	51.0	16.3	29.1	11.7	36.8	13.6	40.8	19.5	11.7–51.0	13.6–40.8	27.0	27.7	0.65
a	− 0.6	21.4	0.4	14.2	24.0	11.1	3.8	18.0	− 0.6 to 21.4	3.8–24.0	8.8	14.2	1.21
b	2.5	− 2.5	2.1	− 2.4	10.1	4.4	19.0	5.2	− 2.5 to 2.5	4..4–19.0	− 0.1	9.7	− 30.21
Hue	− 76.0	− 6.9	77.5	− 10.6	23.1	21.5	78.5	16.3	− 76.0 to 77.5	16.3–78.5	− 4.0	34.8	− 15.76
Chroma	2.5	21.5	2.1	14.4	26.0	12.0	19.4	18.7	2.1–21.5	12.0–26.0	10.1	19.0	0.94

*DGF1 = *Hylocereus undatus*; DGF2 and DGF4 = *Hylocereus costariscensis*; DGF3 = *Hylocereus megalanthus*.

**TPC = Total phenol content (mgGAE 100 g^−1^); TFC = Total flavonoid content (mgRE 100 g^−1^); TCC = Total carotenoid content (µg g^−1^); β-Carotene (µg 100 g^−1^); Xanthophyll (µg g^−1^).

### Molecular characterization

**Figure 4:**
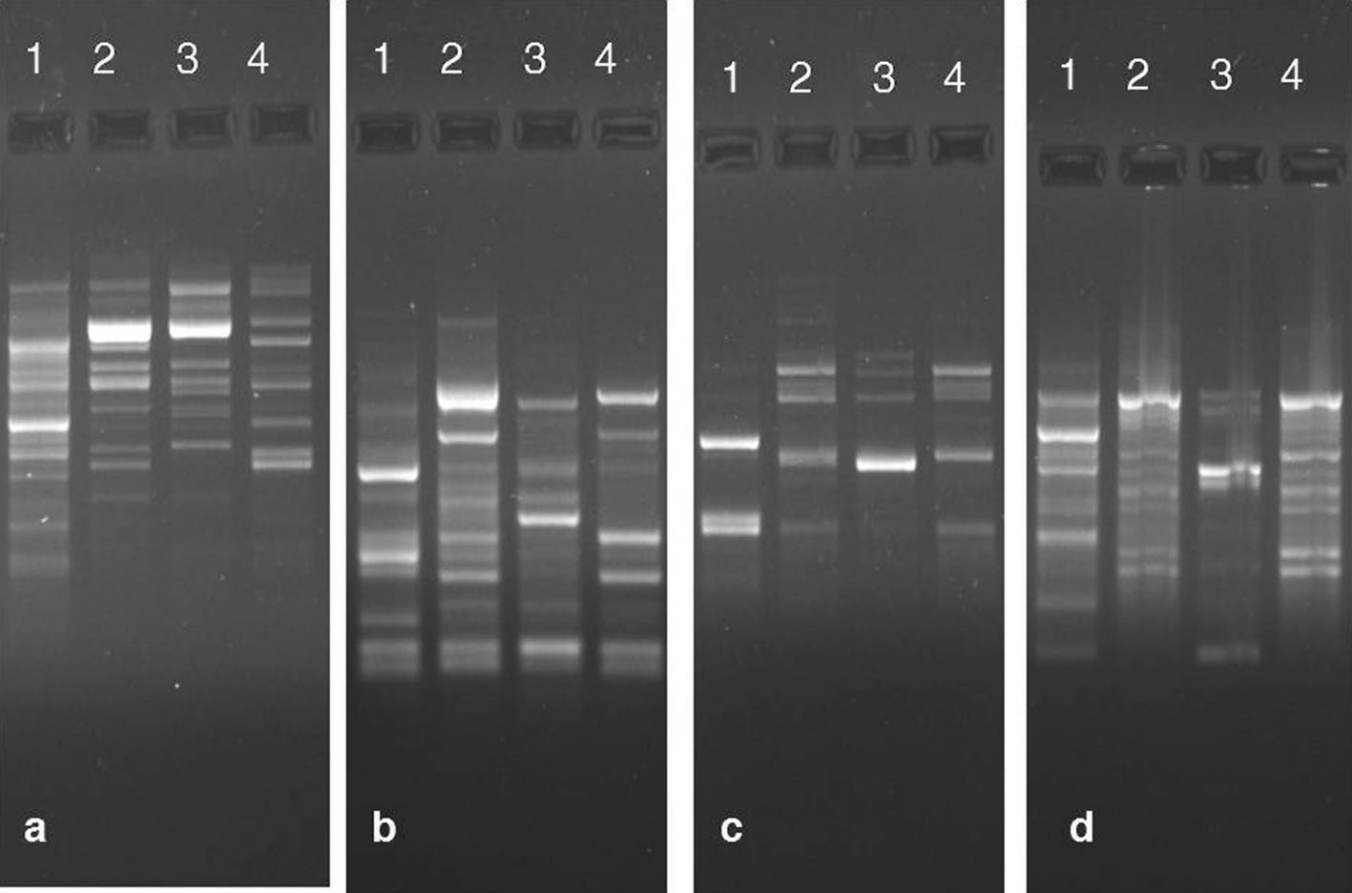
ISSR marker profiles of four dragon fruit genotypes (**a**) UBC900, (**b**) UBC811, (**c**) UBC 824 and (**d**) UBC835. [Lane 1—DGF1 (*H. undatus*); Lane 2—DGF2 (*H. costariscensis*); Lane 3—DGF3 (*H. megalanthus*) and Lane 4—DGF4 (*H. costariscensis*)].

**Figure 5:**
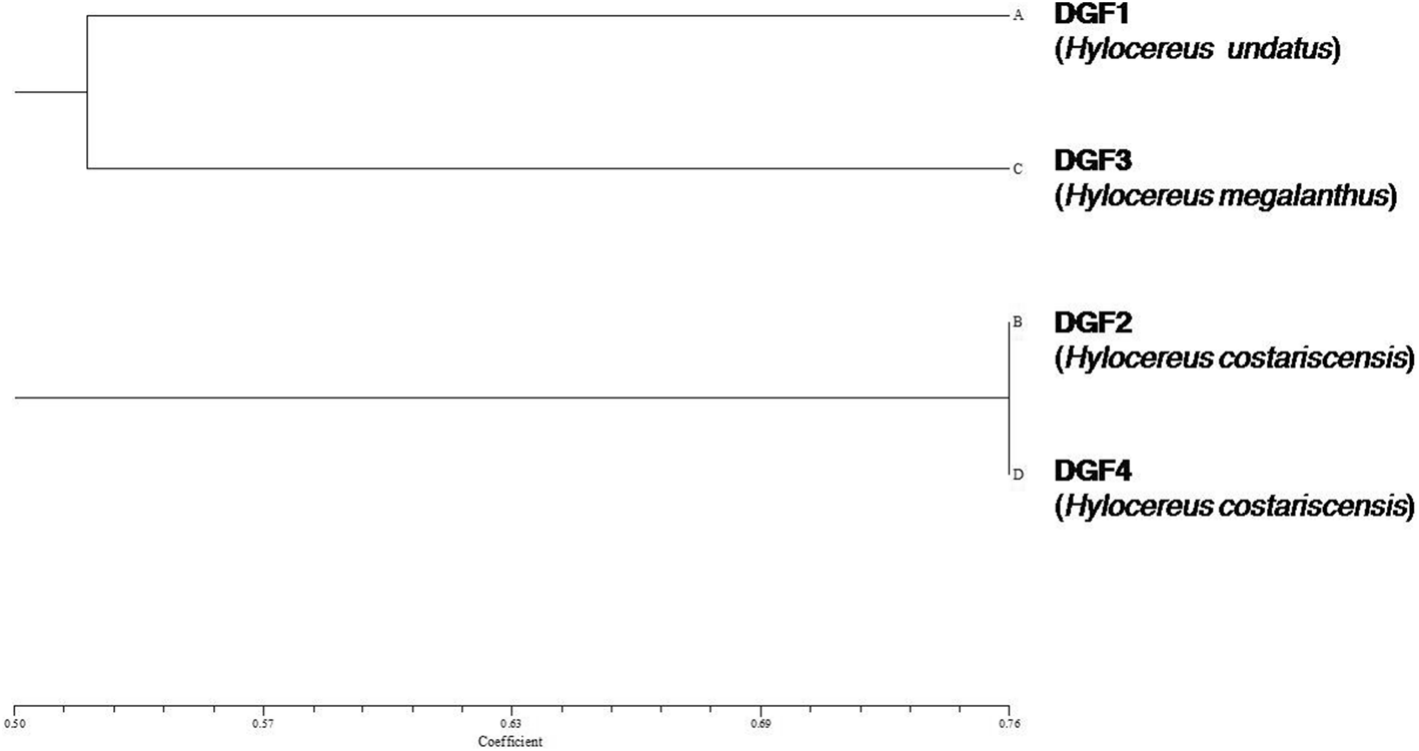
Dendrogram of four dragon fruit genotypes based on UPGMA method of cluster analysis by using Jaccard’s similarity co-efficient obtained from 14 ISSR marker data.

Details of polymorphism obtained for four genotypes of three dragon fruit (*Hylocereus* spp.) species subjected to 16 ISSR marker based genetic diversity study are presented in Table 5. Among 16 ISSR primers screened, 14 primers showed amplification and produced a total of 178 reproducible amplified bands. No amplification was obtained for two ISSR primers viz., UBC825 and UBC853 in all four genotypes, whereas, three ISSR primers viz., UBC815, UBC856 and UBC891 showed no amplification in two genotypes DGF2 and DGF4. The electrophoretic profile of ISSR markers study showed highly distinct and polymorphic banding pattern in primers UBC900, UBC811, UBC824 and UBC835 (Fig. 4). Number of amplified bands varied from 5 in UBC887 to 19 in UBC811 with an average of 12.71 bands per primer. Range of polymorphic bands and % polymorphism observed were 1–13 and 20.0–92.8, respectively. The polymorphic information content value of ISSR marker ranged from 0.42 (UBC895) to 0.91 (UBC 856). Dendrogram was generated by using UPGMA method of cluster analysis that differentiated all four dragon fruit genotypes into two clusters (Cluster I and II) at Jaccard’s similarity coefficient value of 0.50 on the basis of geographical locations of them (Fig. 5). Among them, two genotypes each showed 52% (DGF1 and DGF3) and 76% (DGF2 and DGF4) genetic similarity.

**Table 5:** Genetic diversity analysis of three dragon fruit (*Hylocereus*) species grown under Andaman and Nicobar Island conditions using ISSR markers.

Sl. no.	Primers	Sequence (5′–3′)	T °C	Total number of bands amplified	No. of monomorphic bands	No. of polymorphic bands	% Polymorphism	PIC
1	UBC807	AGAGAGAGAGAGAGAGT	57.0	15	2	13	86.7	0.68
2	UBC810	GAGAGAGAGAGAGAGAT	48.0	14	1	13	92.8	0.66
3	UBC811	GAGAGAGAGAGAGAGAC	55.0	19	6	13	68.4	0.45
4	UBC824	TCTCTCTCTCTCTCTCG	50.0	14	3	11	78.6	0.58
5	UBC835	AGAGAGAGAGAGAGAGYC	60.0	14	4	10	71.4	0.44
6	UBC840	GAGAGAGAGAGAGAGAYT	53.0	15	4	11	73.3	0.45
7	UBC848	CAC ACACACACACACARG	58.0	12	2	10	83.3	0.54
8	UBC880	GGAGAGGAGAGG AGA	55.0	9	2	7	77.8	0.63
9	UBC887	DVDTCTCTCTCTCTCTC	48.0	5	4	1	20.0	0.51
10	UBC895	AGAGTTGGTAGCTCTTGATC	48.0	17	6	11	64.7	0.42
11	UBC900	ACTTCCCCACAGGTTAACACA	50.0	14	3	11	78.6	0.48
12	UBC815*	CTCTCTCTCTCTCTCTG	56.0	9	0	6	66.6	0.88
13	UBC856*	ACACACACACACACA CYA	55.0	13	0	11	84.6	0.91
14	UBC891*	HVHTGTGTGTGTGTGTG	50.0	8	0	4	50.0	0.84
15	UBC825^+^	ACACACACACACACACT	54.0	–	–	–	–	–
16	UBC853^+^	TCTCTCTCTCTC TCTCRT	52.7	–	–	–	–	–
Sum				178	37	132	–	–
Average				12.7	2.64	9.43	71.2	–
Range				5–19	0–6	1–13	20.0–92.8	0.42–0.91

R = (A or G), Y = (C or T), D = (A, G or T), H = (A, C, T), V = (A, C or G).

UBC, ISSR primers, designed from University of British Columbia, Vancouver, Canada.

## Discussion

Dragon fruit (*Hylocereus* spp.) is a promising tropical fruit which can be cultivated in different tropical and subtropical parts of the world such as Southeast Asia and Central and South America. Its health benefits to human can be explained by its essential nutrients such as vitamins, minerals, complex carbohydrates, dietary fibres and antioxidants. Dragon fruit is also an essential source of betacyanin which serves as a red/ purple pigment with anti-oxidative properties. Morphological and genetic heterogeneity on fruit and other characters formed over the period in dragon fruit by high intra- and inter-specific hybridization made taxonomical confusion to identify them at species level. So, the present study was undertaken to identify the key traits in dragon fruit (*Hylocereus* spp.) using morphological, biochemical and molecular (ISSR marker) characterization for distinguishing them at species level.

Thirty four quantitative and 26 qualitative traits of four dragon fruit genotypes belonging to three different species subjected to morphological characterization showed presence of considerable amount of genetic variations among them especially for fruit characters such as fruit weight, pulp weight, number of fruiting cycles, fruit yield, fruit shape, peel and pulp colour. In case of qualitative traits, all three species showed light reddish young cladode colour, edged sepal pattern, ovate shape of pericarpel bracts and milky white petals in flower. Traits such as cream coloured floral stigma lobe and medium sized seed in fruit with broad to medium fruit width of *H. costariscensis* could be useful in taxonomic aspects to differentiate the species with others. Cladode, floral and fruit characters of *H. megalanthus* such as margin ribs of cladode and its waxiness; sepal colour, colour of ring at base of reproductive organs in flower; fruit shape, position towards peel, pulp colour, peel colour and seed size in fruit are visible taxonomic traits to distinguish this species with other two *Hylocereus* spp., *H. undatus* and *H. costariscensis*.

Cladode characters such as cladode width (mm), distance between areoles (mm), number of spines, length of areoles (mm), margin ribs of cladode and its waxiness could be used for identification of *Hylocereus* spp. ^18,19^, as *H. megalanthus* showed 35, 40, 3, 1, concave and light waxy respectively, as compare to other species [*H. undatus* (39, 38, 4, 3, convex and weak) and *H. costariscensis* (38–53, 34–35, 4 or 5, 4 or 1, convex and weak or strong white wax)] in our study. Values of cladode width and distance between areoles was corresponding well with earlier study from Mexico (Castillo-Martínez et al.^20^), who obtained as 42–54.3 and 35–50 mm, respectively.

Floral traits of *H. costariscensis* such as flower length (cm), length of style (cm), number of stigma lobes and bifurcation of stigma lobes (cm) were comparatively lower (as 18.36–19.26, 7.02–7.34, 20.3–23.4 and 1.67–1.76) than other two species [*H. undatus* (23.2, 13.4, 28.2 and 2.18) and *H. megalanthus* (22.15, 12.89, 28.1 and 2.13)]. Flower length (cm) obtained for three species (18.3–23.2) was lower than earlier studies [28.6–34.1 by Tran and yen^21^; 30.0–37.0 by Dios^22^; 23.0–28.0 by Castillo-Martínez et al.^20^]. Natural flowering and production occurs during warmer months in dragon fruits^23,24^ and the flowering season also varied between May to October across different regions of the world. Though flowering and fruiting occurs between April to November under Island condition, much variations were observed in flowering, fruiting period and fruiting cycles among three species.

Fruit morphology such as size and colour of fruit is the main taxonomic evidences to differentiate among several *Hylocereus* spp. and also exhibits the external quality of fruit^22^. Colour of peel and pulp of fruit identified as one of the main key traits to differentiate the three different dragon fruit species as pinkish green peel with white pulp (*H. undatus* -DGF1), pink/ pinkish red peel with pink/ dark purple pulp (*H. costariscensis* -DGF2 and DGF4) and yellow peel with white pulp (*H. megalanthus* -DGF3) (Fig. 1). Maximum fruit set percentage (83.8%) observed for *H. undatus* might be due to its self-compatible nature^25^ compare to other two species. The presence of high number of natural polliantors such as hawk moths and bats during night hours in the field is playing major role in fruit set in dragon fruit^26^, whereas, only honey bees observed to be the pollinators during early morning hours under Island condition. Therefore, introduction of hawk moths could ensure natural pollination and also artificial pollination may aid in increased fruit set percentage in dragon fruit genotypes. Further, the low average fruit yield of *H. megalanthus* -DGF3 and *H. costariscensis*—DGF4 could directly be linked to their number of fruiting cycles as 1 and 2, respectively with comparison to *H. undatus*—DGF1 (7) and *H. costariscensis*—DGF2 (8). Total soluble solids, being the most desirable character in view of consumers’ preference, measured as °Brix, which can be affected by a set of factors such as genetic, climatic, soil, management, among others^27–29^. In the present study, the TSS ranged between 9.1 to 18.3^o^B representing better fruit quality which evidenced by the earlier report that the TSS values between 11 to 15^o^B have good market preference^30^.

Phenolic compounds are playing vital role in multiple biological activities such as anti-mutagenicity, anti-carcinogenicity, anti-aging and also anti-oxidant in plant^31^. Phenolic acid (e.g. gallic acid) and polyphenol (e.g. flavonoids) are highly correlated with antioxidant activity as evidenced from earlier studies^32^. Phenol and flavanoid content at peel and pulp of fruit varied with low (*H. undatus*) to medium (*H. megalanthus*) and with medium/ high (*H. costariscensis*) could be used as taxonomic purposes to distinguish species at bio-molecular level. The present study revealed that inedible peels of dragon fruit had higher phenolic content as compared to edible pulps. Comparatively, higher amount of total phenolic content (mg GAE 100 g^−1^) obtained in present study (32.5–130.0 and 71.0–161.3 in pulp and peel, respectively) than earlier studies such as Nurliyana et al.^9^ for peel and pulp as 28.2 and 19.7 for *H. undatus* and as 36.1 and 3.8 for *H. polyrizhus*; Nurul and Asmah^33^ for dragon fruit juice from Malaysia (70.2) and Australia (72.8).

Huge amount of differences in phenolic content obtained among four dragon fruits in the present study might be due to defend against or mitigate them from adverse effects of biotic and abiotic stresses of sub-tropic climate^34^. Further, high phenolic content of dragon fruit could definitely be a good source of polyphenol to be integrated into the human diet compare to common fruits^35^. Similar kind of results on phenol and flavonoid content of our study was reported by Ramli et al.^36^ who obtained phenol content as 73.8 and 121.8 and flavonoid content as 145.9 and 510.7 in pulp and peel fractions, respectively. This might be due to the fact that non-flavonoid and flavonoid compounds found in pulps and peels, respectively^37^. Comparatively, the presence of high phenol and flavonoid content in peels than pulp of dragon fruit indicating higher antioxidant potential of peel extract in quenching free radicals.

Generally, carotenoid protects the plant from photo oxidation^38^ which is evidenced from its essential role in chloroplasts and chromoplasts^39^ and as xanthophylls in chlorophyll^40^. Whereas, human bodies are also able to transform dietary carotenoids to biologically active vitamin A (retinol) and its derivative, one RE (retinol equivalent) corresponds to either 6 μg of dietary β-carotene or 12 μg of other dietary pro-vitamin A^41^. Dragon fruits identified with high carotenoid content such as *DGF4 and DGF2* (*H. costariscensis*) than other two species could be an excellent source of provitamin A, because 200 g of this fruit (pulp and peel) would meet the daily vitamin A requirement of 700 and 900 μg day^−1^ for adult women and men, respectively^42^. So, these two genotypes may be having potential for the development of nutraceutical products to meet out the vitamin-A deficiency among humans in tropical regions.

Being most powerful scavenger of singlet oxygen, β-carotene’s lower plasma levels can even lead to death^43^. In present study, the genotype with highest content of β-carotene DGF4 (55.86 µg 100 g^−1^) with dark purple pulp is having nearly 50 times more than white pulp fruits. Further, these white pulp fruits (DGF1 and DGF3) were low in xanthophyll content also. Therefore, the dragon fruit genotype ‘DGF4′ could be an alternate good source for many common fruits such as apple (24), grape (39), Kiwi fruit (18)*,* papaya (32) and pepper (44)^44^ and under-utilized fruits such as durian fruit (23)^45^, jack fruit (22) and guava (1.0)^46^. High carotenoids and xanthophylls content in DGF4, DGF2 and DGF3 may be due to high amount of chlorophyll synthesized in chloroplast as plant pigments which is responsible for colour of different capacity^47^.

The DPPH based scavenging activity (%) of four dragon fruit genotypes revealed the higher scavenging activity of peels (55.6–81.2) than pulp (36.0–75.3) extracts under present study. This values were not exactly corresponded well to levels of total phenol and flavonoid contents in both pulp and peel fractions of fruits. This non-significant differences observed in DPPH activity among genotypes, except DGF4 might be due to (1) the presence of lypophilic compounds in the fruits for TPC, (2) over-estimation of TPC by Folin–Ciocalteu reagent method^48,49^, (3) varying response of the Folin–Ciocalteu method to different phenolic compounds^50,51^ and (4) removal of non-phenolic compounds (flavonoids) having antioxidant capacity during methanolic extraction. Further, the high TPC with low antioxidant capacity (AC) as observed under present study was also reported earlier in some underutilized fruits such as *Garcinia*, *Nephelium* and Syzygium fruits^52^.

Negative correlation between either phenolic or flavonoid content and ABTS activity observed under present study was also reported in earlier^53,54^. This variations could be due to (1) difference in number of phenolic groups of polyphenolic compounds which led to differently response to ABTS activity of pulp fraction^55^, and/ or (2) presence of high amount of reducing agents such as ascorbic acid, minerals and carotenoids in the fruits^48,49^, high protein content or genetic, agronomic and environmental influences^56^. In case of flavonoid compounds, both peel and pulp extract had strong influence on only DPPH activity which means that extracts had higher ability in absorbing H^ +^ ions to form stable radicals rather than electrons (ABTS activity). Only β-carotene content of dragon fruit genotypes were moderately correlated with both the DPPH and ABTS based scavenging activity. Comparatively, ABTS based scavenging activity (%) found highest in DGF2 (87.9 and 89.8) and moderate in DGF1 (68.1 and 61.8) and DGF3 (56.4 and 82.4) in pulp and peel, respectively, was more than DPPH based scavenging activity. It might be due to the difference in scavenging activity of both DPPH and ABTS method that former one is based on hydrogen atom transfer (HAT) only, whereas the later one is based on both hydrogen and electron transfer (ET) and also to ABTS radical’s more sensitivity to phenolic-containing compounds than DPPH one.

Colour difference between pulps of the three different *Hylocereus* species was clearly distinguished by their fruit colour values such as high ‘L’ value for white coloured pulp [51.0 in DGF1 (*H. undatus*) and 29.1 in DGF3 (*H. megalanthus*)], high ‘a’ value for pink or dark purple coloured pulp [21.4 in DGF2 (*H. costariscensis*) and 14.2 in DGF4 (*H. costariscensis*)], high ‘b’ value for yellow coloured peel (19.0 in DGF3). Betalains (red violet ‘betacyanin’ and yellow ‘betaxanthins’) are water soluble pigments that provide colours in flowers and fruits^8^. The white pulped fruits with low phenolic content might be due to non-betalainic phenolic compounds which could lead to lower radical scavenging activity of them and the major antioxidant capacity of pink or purple pulped fruits were due to the presence of betalains^57^.

Morphological characterization along with molecular characterization using ISSR markers would provide strong base for unravelling the genetic diversity between the different genotypes of dragon fruit. ISSR profiling is efficient to reveal the genetic diversity in many crops^58–60^. In dragon fruit, utilization of ISSR markers were first reported in china by Tao et al.^61^ to elucidate the genetic relationship of red pulp genotypes from white pulp ones. Range of number of polymorphic bands (1–13) and % polymorphism (20–92.8%) obtained in this study was almost comparable with results of Tao et al.^61^ who obtained as 1–7 and 25–100%, respectively, on 50 red and white pulped dragon fruit accessions in China. Like this, the other PCR based marker systems such as RAPD (Legaria Solano et al.^62^ in Mexico; Junqueira et al.^63^ in Brazil; Rifat et al.^64^ in Bangladesh) and AFLP (Pagliaccia et al.^65^ in USA) markers were also showed high amount of % polymorphism on dragon fruit accessions grown in different regions of world. Comparable PIC value of ISSR markers (0.49–0.92) reported by Tao et al.^61^ as in our study (0.42–0.91). Dendrogram obtained for four dragon fruit genotypes clearly differentiated the genus *Hylocereus* at species level on the basis of their geographic origin and pulp colour by clustering dark purple or pink pulped species, *H. costariscensis* (DGF2 and DGF4) as one group and the other two white pulped species, *H. undatus* (DGF1) and *H. megalanthus* (DGF3) in another group. It indicated the effectiveness of PCR based dominant ISSR marker for species differentiation and quantification of genetic diversity as evidenced in earlier studies on plants^59,66,67^.

## Conclusion

Morphological, biochemical and molecular characterization of four dragon fruit (*Hylocereus* spp.) genotypes grown in Andaman and Nicobar Island revealed the presence of considerable amount of genetic variations among them which could be used as key traits for distinguishing three different species under present study. Cladode and fruit characters showed higher variability among morphological traits. Comparatively, the presence of high phenol and flavonoid content in peels than pulp of fruit indicating higher antioxidant potential of peel extract in quenching free radicals and was evidenced by higher DPPH-based scavenging activity of peels than pulp extracts. Comparatively, ABTS-based scavenging activity (%) found highest in DGF2 (87.9 and 89.8) and moderate in DGF1 (68.1 and 61.8) and DGF3 (56.4 and 82.4) in pulp and peel, respectively was more than DPPH-based one. ISSR-marker based clustering pattern clearly differentiated the genus *Hylocereus* at species level on basis of their geographic origin and pulp colour by grouping them separately. Key traits identified to differentiate three different *Hylocereus* species were: Pulp/ peel colour of fruits, number of spines and length of areoles in cladode, colour values and phenol/ flavanoid contents of fruits. Genotypes with high carotenoid and xanthophylls content (DGF4 and DGF2) and identified in the study may be having potential for development of nutraceutical products to meet out vitamin-A deficiency among humans in tropical regions.

## Materials and methods

### Experimental materials

Among the seven Dragon fruit (*Hylocereus* spp.) germplasm collections maintained at the experimental field of Garacharma farm (11° 36′ 392′′ N; 092° 36′ 037′′ E) located at ICAR-Central Island Agricultural Research Institute, Port Blair, Andaman and Nicobar Islands (India), four genotypes belonging to three species such as *H. undatus* (DGF1), *H. costariscensis* (DGF2, DGF4) and *H. megalanthus* (DGF3) were selected based on their growth, flowering and fruiting behaviour under island conditions for present study (Fig. 1). The native origin of *H. costariscensis* is Costa Rica and Nicaragua, whereas *H. megalanthus* is from northern South America. Although all true cacti originated in the Americas, the precise origin of *H. undatus* is uncertain owing to its hybrid nature.

### Morphological characterization

Ten plants per species/ genotype were used for morphological characterization and the observation made on 34 quantitative and 26 qualitative traits which include cladode, flowering and fruit characters chosen from descriptors of IBPGR/ NBPGR for three consecutive years from 2015–2016 to 2017–2018 under open field conditions (Tables 1 and 2).

### Biochemical characterization

Fresh matured fruits of four genotypes belonging to three dragon fruit species namely DGF1, DGF2, DGF3 and DGF4 were harvested and washed with millipore water. Peel and pulp of fruit samples were separated for further analysis of biochemical parameters viz., phenols, flavanoids, carotenoids and antioxidants including fruit colour values and the details of methods used (Supplementary file 1). A Hunter-Lab Colorimeter (MiniScan XE Plus 4500 L) was used for fruit colour measurement (peel and pulp). In case of phytochemicals, the methods used were: (1) for total phenolic content (TPC), the Folin-Ciocalteu colorimetric method^68^, (2) for total flavonoid content, a colorimetric method^69^, (3) for total carotenoid content^70^, (4) for β-carotene and xanthophyll^71^, (5) for free radical scavenging activity (RSA), total antioxidant activity by DPPH^72^ and ABTS^55^ methods.

### Molecular characterization

DNA samples were isolated from stem tissue of four dragon fruit genotypes using a modified CTAB method^73^. The quality and concentration of the DNA were confirmed by electrophoresis on 1% agarose gels. Selection of primers of Inter Simple Sequence Repeats (ISSR) marker were done to analyze genetic variation among four genotypes^61^ and their details of primer sequence and annealing temperature are listed in Table 5. PCR reaction mixtures were prepared in a final volume of 10 μl, containing 10 µl volume containing 1 µlDNA (20 ng/µl), 5.0 µl PCR mix (Qiagen), 0.8 μl primer and 3.2 μl nuclease-free water and PCR amplification were performed in C1000 Touch Thermal Cycler (Bio-Rad) with an initial denaturation step of 5 min at 94 °C, followed by 40 cycles of denaturation for 45 s at 94 °C, annealing for 45 s at primers specific temperatures and extension for 1 min at 72 °C, and ended with a final extension step of 5 min at 72 °C. Banding pattern from each primers were resolved in 2% agarose gels using Gel Doc XR + Imager (Bio-Rad) and all reactions were repeated thrice to confirm the consistency in banding pattern before subjected to scoring.

### Statistical analysis

Data observed on 34 quantitative morphological traits of dragon fruit genotypes were subjected to basic statistical analysis using software PAST 3^74^. Molecular data were observed on basis of banding patterns by scoring the presence of band as ‘1′ and absence as ‘0′ for particular fragment in lane. Polymorphism information content (PIC) was calculated following Weir^75^ method according to the formula: PIC_i_ = 1 − ∑(P_ij_)^2^, where, Pij is the frequency of the jth locus for the ith marker and is summed over i loci/marker. The Un-weighted Paired Group Method with Arithmetic means (UPGMA) based Jaccard's Similarity Coefficient was used for cluster analysis. This computation was performed by using NTSYS‐pc v2.2^76^.

### Ethical standards

The authors declare that there is no ethical issue(s) in this study.

## Supplementary Information

Supplementary Information 1.
