# Supplementary material for: Distinguishing three Dragon fruit (Hylocereus spp.) species grown in Andaman and Nicobar Islands of India using morphological, biochemical and molecular traits
Source: Sci Rep. 2021 Feb 3;11:2894. doi: 10.1038/s41598-021-81682-x (PMC7859243; doi:10.1038/s41598-021-81682-x)
Supplement: Supplementary file 1 — Supplementary Information 1. [file 41598_2021_81682_MOESM1_ESM.pdf]

Distinguishing three Dragon fruit (*Hylocereus* spp.) species grown in Andaman and Nicobar Islands of India using Morphological, Biochemical and Molecular traits

Author's list with affiliations:

1) **K. Abirami,** Senior Scientist (Fruit Science), Division of Horticulture and Forestry, ICAR- Central Island Agricultural Research Institute, Port Blair - 744 101, Andaman and Nicobar Islands, India**.** e-mail: abirami78@gmail.com
2) **S. Swain,** Scientist (Agricultural Structure and Process Engineering), Division of Natural Resource Management, ICAR- Central Island Agricultural Research Institute, Port Blair - 744 101, Andaman and Nicobar Islands, India. **Present address**: ICAR- Central Institute for Women in Agriculture (CIWA), Bhubaneshwar – 751 003, Odisha, India. E-mail: sachi9463@gmail.com
3) **V. Baskaran,** Principal Scientist (Floriculture and Landscaping), Division of Horticulture and Forestry, ICAR- Central Island Agricultural Research Institute, Port Blair - 744 101, Andaman and Nicobar Islands, India**.** E-mail: vbaski01@gmail.com
4) **K. Venkatesan,** Scientist (Economic Botany), Division of Field Crop Improvement and Protection, ICAR- Central Island Agricultural Research Institute, Port Blair - 744 101, Andaman and Nicobar Islands, India. Corresponding author e-mail: venkipgr08@gmail.com
5) **K. Sakthivel,** Scientist (Plant Pathology), Division of Field Crop Improvement and Protection, ICAR- Central Island Agricultural Research Institute, Port Blair - 744 101, Andaman and Nicobar Islands, India. **Present address**: ICAR- Indian Institute of Oilseeds Research, Hyderabad – 500 030, Telangana, India. E-mail: veluars@gmail.com
6) **N. Bommayasamy,** Subject Matter Specialist (Agronomy), Krishi Vigyan Kendra, ICAR- Central Island Agricultural Research Institute, Sipphighat, Port Blair - 744 101, Andaman and Nicobar Islands, India. E-mail: samygs81@yahoo.co.in

Colour measurement

Colour measurement of peel and pulp of four accessions were carried out using a Hunter-Lab Colorimeter (MiniScan XE Plus 4500 L). The instrument ($45^o/0^o$ geometry, D 65 optical sensor, 10° observer) was calibrated with black and white reference tiles through the tri-stimulus values X, Y, Z, taking as standard values those of the white background (X=79.01; Y=83.96; Z=86.76) tile. The colour values were expressed as L (whiteness or brightness/ darkness), a (redness/greenness) and b (yellowness/blueness) at any time respectively. Color measurements were taken in triplicate and average values were taken for calculation. The derived colour parameters chroma and hue angle were computed as chroma = $(a^2+b^2)^{0.5}$, Hue = $\tan^{-1}(b/a)$. Chroma represents 'richness of colour' or colour intensity, while hue angle depicts how an average person will perceive that colour.

Total phenolic content (TPC)

The Folin-Ciocalteu colorimetric method was used to measure the total phenolic content (Singleton et al., 1999) with minor modifications. Briefly, 200 µl of the extractions were oxidized with 1 ml of 0.5 N Folin-Ciocalteu reagent and then the reaction was neutralized with 1 ml of the saturated sodium carbonate (75 g/L). The absorbance of the resulting blue color was measured at 760 nm with a UV-VIS Spectrophotometer, UV-2600 (Shimadzu, Japan) after incubation for 2 h at room temperature. Quantification was done on the basis of the standard curve of gallic acid. Results were expressed as milligram of gallic acid equivalent (mg GAE) per 100 g of flour weight.

Flavonoid content

Total flavonoid content was determined by a colorimetric method (Bao *et al*., 2005). 0.5 ml extracts were added to 15 ml polypropylene conical tubes containing 2 ml ddH_2O and mixed with 0.15 ml 5% $NaNO_2$. After reacting for 5 min, 0.15 ml 10% $AlCl_3.6H_2O$ solution was added. After another 5 min, 1 ml 1 M NaOH was added. The reaction solution was well mixed, kept for 15 min and the absorbance was determined at 415 nm. Qualification was done using the Rutin as standard and the result was expressed as milligrams of rutin equivalent (mg QE) per 100 g of dry weight.

Determination of total carotenoids, β-carotene and xanthophyll

Total carotenoid content was determined by the method described by Sadasivam and Manikam, 1994 with minor modifications. Fresh sample (2 g) ground well in 10 ml of acetone and centrifuged at 5000rpm for 8min in a cooling centrifuge at 4^oC. Supernatant was repeatedly ground and filtered till devoid of the color. The solvent was removed by rotary evaporator. The extract was transferred to a 50ml volumetric flask containing 15 g of anhydrous sodium sulphate. The volume was made up by petroleum ether and the absorbance read at 450 nm.

Total carotenoids were calculated as follows and expressed as milligrams per g of dry weight (DW).

$$\text{Total carotenoids } (\mu g/g) = (A \times V \times 10^6) / A^{\%} \times W$$

A is the absorbance at 450 nm, *V* is the total volume of extract (ml), $A^{\%}$ is the extinction coefficient (2592 cm for petroleum ether) and W is sample weight (g).

β-carotene (µg/100 g) and xanthophyll (µg/g) were determined spectrophotometrically at 470, 645, and 662 nm, respectively and their content were estimated using the equations described by Lichtenthaler and Buschmann (2001) as follows: β-carotene = 1000A470 −2.270 Ch 'a' −81.4 Ch 'b' 227 where Cha = Chlorophyll a, and Chb = Chlorophyll b. Xanthophyll determined by subtracting β-carotene fraction from total carotenoids.

Free radical scavenging activity (RSA)

Total antioxidant activity was measured both by DPPH and ABTS methods.

DPPH (2,2- diphenyl-1-picrylhydrazyl) activity

Similarly, total antioxidant activity was obtained by 2,2-diphenyl-2-picrylhydrazyl (DPPH) method (Rattanachitthawat *et al*., 2010) with some modification. The working solution of DPPH was freshly prepared by diluting 3.9 mg of DPPH with 95 % ethanol to get with an absorbance of 0.856 ± 0.05 at 517nm. The different concentration of extract was mixed with 1.5 ml of working DPPH and the absorbance of the mixture immediately measured spectrophotometrically after 10 min. Total antioxidant activity of the extracted sample extract was expressed as mg BHA/ g sample equivalent, obtained from the calibration curve.

% inhibition of DPPH radical = $(A_{control} - A_{sample}/A_{control}) \times 100$

Where $A_{control}$ is the absorbance of the control (without extract) and A_{sample} is the absorbance in the presence of the extract/standard.

ABTS (2, 2'-azino-bis (3-ethylbenzothiazoline-6-sulphonic acid) activity

The total antioxidant capacity was determined by a colorimetric method (Re et al., 1999) with a little modification. The ABTS radical cation was generated by oxidation of 7 mM ABTS with $K_2S_2O_8$ (2.45 mM) in 10 mL of deionzed water and kept in darkness at 4 °C during 16 hours. Once the radical cation was obtained, the $ABTS^+$ cation solution was diluted with 80% ethanol to an absorbance of 0.784±0.01 at 734 nm. Then, 3.9 ml $ABTS^+$ cation solution was added to 1 ml of extracts and mixed thoroughly. The mixture incubated for 6 min at room temperature and tested the absorbance at 734 nm. Results were expressed in terms of Trolox equivalent antioxidant capacity (TEAC, mM Trolox equivalents per 100 g dry weight).

% inhibition of ABTS radical = $(A_{control} - A_{sample}/A_{control}) \times 100$
